# Risk factors of diastasis recti abdominis, and relationship with surface electromyography characteristics of pelvic floor muscles in early postpartum: a retrospective study

**DOI:** 10.7717/peerj.20299

**Published:** 2025-11-07

**Authors:** Xiaojun He, Yang Lin, Sha He, Juan Lin

**Affiliations:** Department of Women’s Health Care, Fujian Maternity and Child Health Hospital, College of Clinical Medicine for Obstetrics & Gynecology and Pediatrics, Fujian Medical University, Fuzhou, Fujian, China

**Keywords:** Diastasis recti abdominis, Pelvic floor muscles, Surface electromyography, Risk factors

## Abstract

**Objective:**

This study aimed to identify the factors associated with diastasis recti abdominis (DRA) in early postpartum women and investigate any relationship with surface electromyography (sEMG) characteristics of pelvic floor muscles (PFM).

**Methods:**

A total of 478 participants who visited Fujian Maternity and Child Health Hospital for postpartum re-examination between January and March 2023 were divided into two groups: DRA and Non-DRA. Basic demographic data were collected *via* self-reported questionnaires. Additionally, inter-recti distance (IRD) was measured using ultrasound imaging, and pelvic floor muscle activity was assessed using surface electromyography according to the Glazer protocols.

**Results:**

There were no significant differences between the non-DRA and DRA groups in terms of weight gain during pregnancy, physical activity, number of fetuses, delivery mode, gestational diabetes, or urinary incontinence during pregnancy or postpartum. However, the DRA group was older and had a significantly lower level of education. Both pre-pregnancy and postpartum body mass index (BMI) were higher in the DRA group. The proportion of first-time mothers was greater in the non-DRA group, and fetal weight was lower in the non-DRA group compared to the DRA group. Multivariate logistic regression analysis revealed that older maternal age, lower education level, and a high number of deliveries were independently associated with an increased risk of DRA. No significant differences were observed in sEMG parameters between the two groups at the pre-baseline, flick contraction, tonic contraction, endurance contraction, and post-baseline stages.

**Conclusion:**

Older maternal age, lower educational attainment, and higher parity were significantly associated with an increased risk of diastasis recti abdominis in early postpartum. No correlation was found between the sEMG characteristics of pelvic floor muscles and diastasis recti abdominis in the early postpartum period.

## Introduction

Diastasis recti abdominis (DRA) is defined as the separation of the two rectus abdominis muscles by more than two cm, resulting from thinning and widening of the linea alba (Hernández-Granados et al., 2021). This condition is common during pregnancy as the abdominal wall expands to accommodate the growing fetus. Previous studies have reported that the prevalence of DRA ranges from 27% to 100% during the second and third trimesters of pregnancy and from 30% to 68% in the postpartum period (Boissonnault & Blaschak, 1988; Fernandes et al., 2015). As a crucial component of the abdominal core, the rectus abdominis plays a vital role in posture maintenance, trunk and pelvic stabilization, respiration, and visceral support.

The pelvic floor muscles (PFM), which are frequently affected during the postpartum period, are equally important for pelvic health. Composed of multiple muscle layers, including the levator ani group and the coccygeus, the pelvic floor forms a supportive sling at the base of the pelvis (Corton, 2009) and is essential for maintaining continence, supporting pelvic organs, and facilitating sexual function. Pelvic floor dysfunction (PFD) encompasses a spectrum of symptoms such as urinary and fecal incontinence, pelvic organ prolapse, and sexual dysfunction (Rogers et al., 2018).

Importantly, the pelvic floor does not function in isolation. It acts synergistically with the abdominal musculature and the diaphragm to regulate intra-abdominal pressure (Hodges, Sapsford & Pengel, 2007; Sapsford et al., 2001). Evidence suggests that activation of the transversus abdominis and internal oblique muscles occurs during voluntary pelvic floor contraction (Neumann & Gill, 2002), and women with DRA may exhibit a higher risk of stress urinary incontinence, fecal incontinence, and pelvic organ prolapse compared to those without DRA (Spitznagle, Leong & Van Dillen, 2007). Furthermore, co-contraction exercises targeting both the transversus abdominis and PFM have been shown to improve pelvic floor function in women with stress urinary incontinence (Tajiri, Huo & Maruyama, 2014). Nevertheless, other studies report no significant relationship between DRA and pelvic floor dysfunction, suggesting that DRA does not necessarily result in PFM weakness or increased rates of incontinence and prolapse (Bøet al., 2017; Wang et al., 2020; Fei et al., 2021). These conflicting findings underscore the complexity of the interaction between the abdominal wall and pelvic floor muscles.

Surface electromyography was selected in this study because it offers a non-invasive, well-tolerated, and reliable method for quantifying PFM activity, particularly in early postpartum women for whom intramuscular or needle-based recordings are often unsuitable. The use of intravaginal surface electrodes enables direct assessment of neuromuscular activation patterns while minimizing discomfort and facilitating repeated measurements in clinical and research settings.

Given these considerations, this study aimed to investigate the surface electromyography (sEMG) characteristics of the PFM—including vaginal resting pressure, muscle strength, and endurance—in women with and without DRA during the early postpartum period. In addition, we explored the prevalence of urinary incontinence (UI) in both groups. By addressing this knowledge gap, our findings may provide a theoretical basis for optimizing postpartum rehabilitation strategies for women with DRA and PFD.

## Materials & Methods

### Participants

A total of 501 postpartum women were screened who visited Fujian Maternity and Child Health Hospital for postpartum re-examination between January and March 2023. Twenty-three were excluded (incomplete data or history of pelvic surgery), leaving 478 participants for analysis. The inclusion criteria: women between 6 weeks and 2 months postpartum, who underwent PFM sEMG and rectus abdominis distance measurement, and who had not received any prior pelvic floor or rectus abdominis therapy. Exclusion criteria included a history of pelvic surgery, neurological diseases, trunk deformities, or incomplete personal information. The study was granted an informed consent waiver by the Ethics Committee of Fujian Maternity and Child Health Hospital (No. 2024KY219).

### Measurements of the inter-recti distance

**Figure 1:**
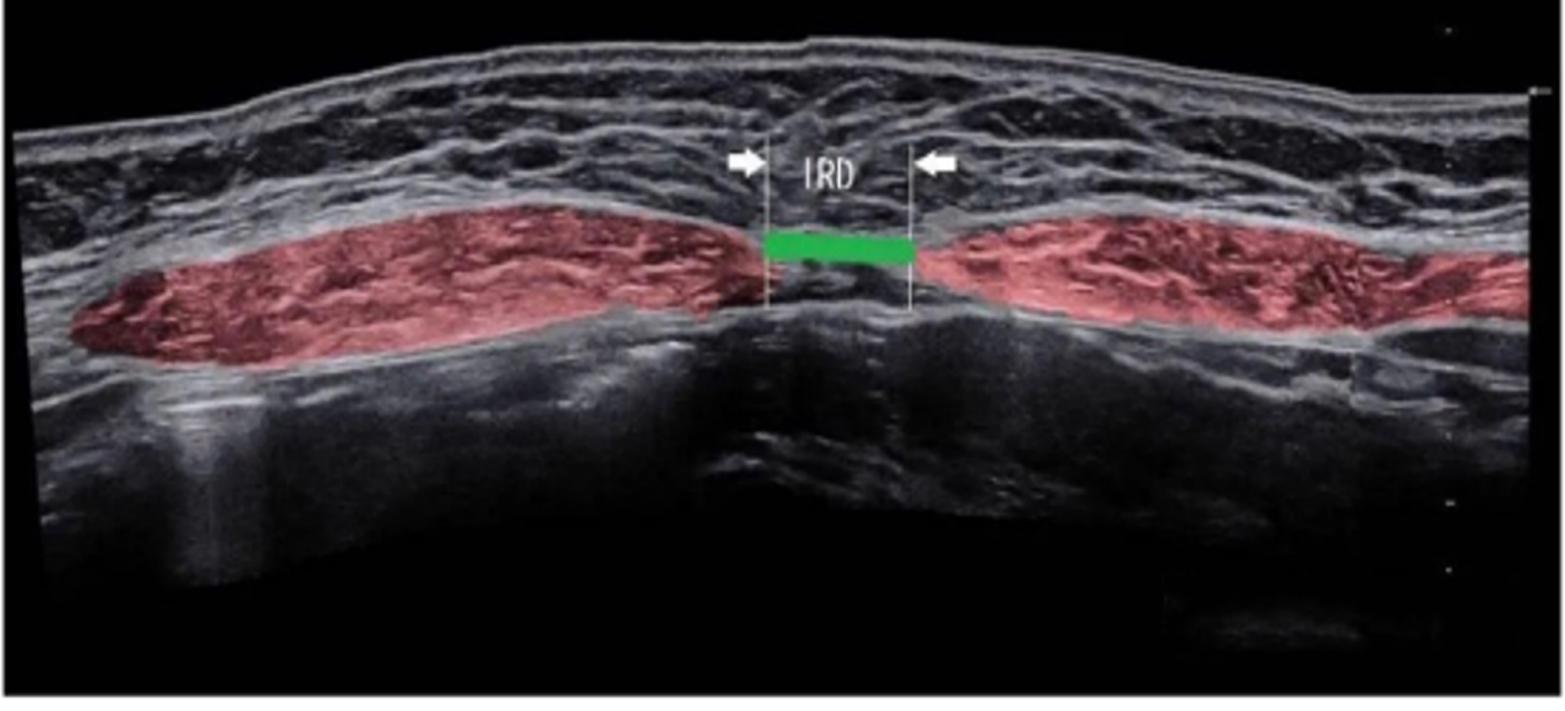
Ultrasound images of diastasis recti abdominis.

The inter-rectus distance (IRD), defined as the width of the linea alba, was measured using ultrasound imaging (PoLinGer, Anhui, China) (Fig. 1). Ultrasound assessments were performed using a high-frequency linear array probe with a frequency of 7.5/10 MHz (probe width: eight cm; 256 elements; electronic scanning). The imaging modes included B-mode, M-mode, color Doppler, and pulsed wave Doppler. The probe was suitable for superficial structures and vascular imaging. The scanning depth was adjustable to ensure optimal visualization of the rectus abdominis muscles and the linea alba. All ultrasound measurements were conducted by a physiotherapist who completed a specialized training program in musculoskeletal ultrasound imaging. This training was organized by the Department of Ultrasound Medicine at Fujian Maternity and Child Health Hospital. The program included over 5 h of theoretical instruction and 20 h of hands-on practical training specifically focused on the evaluation of IRD using B-mode ultrasound. Competency assessment included a written theoretical exam and a practical skills test, both evaluated by senior sonographers with national certification. Measurement standardization was ensured by following the protocol recommended by the European Working Group on Abdominal Muscle Ultrasound, including consistent patient positioning, probe placement at standardized landmarks, and averaging three repeated measurements at each site. Test–retest of ultrasound measurements of IRD demonstrated good to very good reliability, with intraclass correlation coefficient values between 0.74 and 0.90 (Mota et al., 2012).

Measurements were taken at five points along the abdominal midline of the linea alba: 4.5 cm and 3 cm above the umbilicus, 4.5 cm and 2 cm below the umbilicus, and at the upper margin of the umbilical ring (Beer et al., 2009; Chiarello & McAuley, 2013). Participants were positioned supine with both knees bent at 90°. The examination points were marked on the skin while the participants were at rest. The distance between rectus abdominis muscles at rest at the end of expiratory was measured. Subsequently, participants were instructed to cross their hands behind their heads, slowly lift their head and shoulders off the bed while exhaling, and raise their shoulder blades to a 40° angle relative to the bed. This position was maintained for 3 s to ensure proper rectus abdominis contraction (Zhang et al., 2023). Diastasis recti abdominis was classified quantitatively as a separation >2 cm at any of the five anatomical points (Mota et al., 2012).

### Surface electromyography assessments

sEMG assessments were performed by another physiotherapist with over three years of clinical experience in pelvic floor rehabilitation. In addition to clinical experience, the physiotherapist had completed formal training in the Glazer protocol through the Ecole Internationale de Rééducation Périnéale et Pelvienne (EIRPP) International Pelvic Rehabilitation School, and had received European Level 1 Certification for Diagnosis and Treatment of Pelvic Floor Dysfunction. In some studies, the good reliability of qualitative and quantitative assessment of surface electromyography signals has been reported (Navarro et al., 2018; Scharschmidt et al., 2020). Before the evaluation, participants were instructed to empty their bladders and adopt a 120° semi-supine position with knees bent and hips outwardly rotated. A sterile, disposable, two-ring intravaginal electrode probe was inserted into the vaginal canal. Surface EMG signals were recorded using a human biostimulation feedback instrument (MLD B2T, Medlander, Najing, Jiangsu, China) following the Glazer protocol (Glazer & Hacad, 2012), with the signal quality monitored in real-time to confirm appropriate electrode placement and participant compliance. Surface EMG data was filtered using the built-in hardware 1st order high-pass filter set to 10 Hz +/− 10% cut-off. The raw sEMG data were visually checked for artefacts.

The Glazer protocol consisted of the following five phases:

Pre-baseline rest (60 s): Participants were instructed to relax completely, and recorded the electromyographic activity of the pelvic floor muscles in a resting state.

Phasic (Flick) contractions (five repetitions): Participants were asked to rapidly contract and immediately relax their pelvic floor muscles, repeated five times (2-second contraction with a 2-second rest between contractions).

Tonic contractions (five repetitions): Participants performed five sustained contractions, holding each contraction for 10 s followed by a 10-second rest period.

Endurance contraction (60 s): Participants were instructed to contract their pelvic floor muscles to a specific level and maintain the contraction for 60 s.

Post-baseline rest (60 s): Participants were again instructed to relax completely, and the post-assessment electromyographic activity of the pelvic floor muscles was recorded.

**Figure 2:**
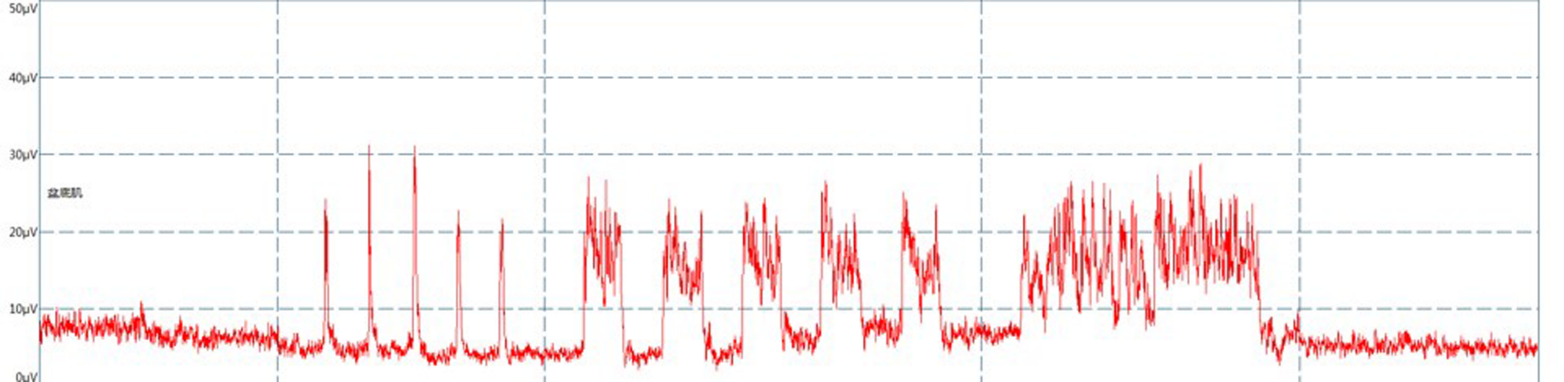
Surface electromyography trace.

The electromyographic trajectory of the pelvic floor muscles will be recorded (Fig. 2).

### Statistical analysis

Statistical analyses were conducted using SPSS software version 25.0 (IBM Corporation, Armonk, NY, USA). Continuous variables were presented as means ± standard deviations if normally distributed, or as medians with interquartile ranges if skewed. Normality was assessed using the Kolmogorov–Smirnov test. For normally distributed data, inter-group comparisons were performed using Student’s *t*-test, while non-normally distributed data were analyzed using the Mann–Whitney *U* test. Categorical variables were expressed as frequencies and percentages, and comparisons were made using the Chi-square test or Fisher’s exact test. A two-tailed *p*-value <0.05 was considered statistically significant. Multivariate logistic regression analysis was performed to identify independent factors associated with diastasis recti abdominis (DRA). Variables with a *p*-value <0.2 in univariate analysis and those with clinical relevance were entered into the regression model. Adjusted odds ratios (ORs) and 95% confidence intervals (CIs) were calculated to assess the strength of associations. A *p*-value <0.05 was considered statistically significant. To control the false discovery rate (FDR) arising from multiple hypothesis testing, we applied the Benjamini–Hochberg (BH) correction to all exploratory comparisons. A result was deemed statistically significant if its *q*-value fell below the FDR threshold of *α* = 0.05. This method ensures that, on average, only 5% of the significant findings are expected to be false positives.

## Results

The study included 478 postpartum women, with a mean age of 30.52 years. Among the participants, 301 (62.97%) were primiparous, and 177 (37.03%) were multiparous. In terms of delivery mode, 337 (70.5%) had vaginal deliveries, while 141 (29.5%) underwent cesarean sections. Diastasis recti abdominis was identified in 347 patients (72.6%), whereas 131 (27.4%) had normal rectus abdominis morphology.

There were no statistically significant differences between the Non-DRA and DRA groups in terms of weight gain during pregnancy, physical activity, number of fetuses, delivery mode, gestational diabetes, urinary incontinence during pregnancy, or postpartum urinary incontinence. However, the DRA group was older than the non-DRA group (95% CI [30.67–31.45], *p* < 0.01). Additionally, the non-DRA group also had a significantly higher level of education, with a greater proportion having higher education compared to the DRA group (*χ*^2^ =23.08, *p* < 0.01). Both pre-pregnancy (95% CI [21.41–22.09], *p* = 0.011) and postpartum BMI (95% CI [23.83–24.44], *p* = 0.016) were higher in the DRA group. The proportion of primiparous women was higher in the non-DRA group (*χ*^2^ = 21.93, *p* < 0.01), while fetal weight was lower in the non-DRA group compared to the DRA group (95% CI [3.20–3.30], *p* = 0.03). After applying the Benjamini–Hochberg (BH) procedure to control the false discovery rate (FDR) across 13 comparisons, the variables above remained statistically significant (Table 1). Multivariate logistic regression analysis revealed that older maternal age (OR = 1.10, 95% CI [1.04–1.16], *p* = 0.002), lower education lever (OR = 0.31, 95% CI [0.18–0.55], *p* < 0.001), and a high number of deliveries (OR = 2.09, 95% CI [1.30–3.37], *p* = 0.002) were independently associated with an increased risk of DRA. Fetal weight, pre-pregnancy BMI and postpartum BMI were not significant factors in the final model (*p* > 0.05) (Table 2).

**Table 1:** Demographic and clinical characteristics.

Characteristics	Non-DRA group (*n* = 131)	DRA group (*n* = 347)	*t*/*Z*/*x*^2^	Cohen’s d/ Cramér’s V	*p*-value	BH-adjusted *q*-value
Age (years)	29.09 ± 4.58	31.06 ± 3.69	−4.69	0.47	<0.01^b^	0.012
Education			23.08	0.22	<0.01^c^	0.015
College/university	35(26.7%)	33(9.5%)				
Primary/high school/other	96(73.3%)	314(90.5%)				
Pre-pregnancy BMI (kg/m^2^)	21.16 ± 2.90	21.75 ± 3.26	−2.4	0.19	0.016^b^	0.021
Weight gain during pregnancy (kg)	13.06 ± 4.71	13.04 ± 5.03	−0.39	0.001	0.695^b^	0.695
Postpartum BMI (kg/m^2^)	23.37 ± 2.95	24.14 ± 2.91	0.24	0.26	0.011^a^	0.022
Physical activity≥1 time per week	29(22.1%)	68(19.6%)	0.38	0.13	0.538^c^	0.592
Number of deliveries			21.93	0.21	<0.01^c^	0.025
1	104(79.4%)	197 (56.8%)				
2	26 (19.8%)	132(38%)				
3	1 (0.8%)	18(5.2%)				
Number of fetuses				0.05	0.565^d^	0.565
1	131 (100%)	344 (99.1%)				
2	0	3 (0.9%)				
Delivery mode			2.04	0.07	0.153^c^	0.218
Vaginal delivery	86 (65.6%)	251 (72.3%)				
Forceps delivery	45 (34.4%)	96 (27.7%)				
Fetal weight (kg)	3.13 ± 0.56	3.25 ± 0.48	−2.17	0.23	0.030^b^	0.030
GDM	20 (15.3%)	56 (16.1%)	0.05	0.05	0.816^c^	0.816
Urinary incontinence during pregnancy	40(30.5%)	112(32.3%)	0.13	0.02	0.715^c^	0.715
Postpartum urinary incontinence	25 (19.15%)	65 (18.7%)	0.008	0.004	0.930^c^	0.930

**Notes.**

BMI body mass index GDM gestational diabetes mellitus

a Student’s *t*-test.

b Mann–Whitney *U* test.

c Chi-square test.

d Fisher’s exact test.

In the tables, *t* represents the test statistic for the independent samples *t*-test, which is used to compare the means between two groups when the data are normally distributed. *Z* refers to the standardized statistic used in non-parametric tests such as the Mann–Whitney *U* test, applied when the data are not normally distributed. *F* denotes the *F*-statistic from one-way analysis of variance (ANOVA), used to compare the means across three or more groups. All statistical tests are accompanied by *p*-values to indicate the level of statistical significance, with *p* < 0.05 considered statistically significant. And *q*-value >0.05 was considered no significant differences after correction.

**Table 2:** Multivariate logistic regression analysis.

Characteristics	Non-DRA group (*n* = 131)	DRA group (*n* = 347)	OR	95%CI	*p*-value
Age (years)	29.09 ± 4.58	31.06 ± 3.69	1.10	1.04–1.16	0.002
Education			0.31	0.18–0.55	<0.001
College/university	35(26.7%)	33(9.5%)			
Primary/high school/other	96(73.3%)	314(90.5%)			
Pre-pregnancy BMI (kg/m^2^)	21.16 ± 2.90	21.75 ± 3.26	0.96	0.85–1.09	0.55
Postpartum BMI (kg/m^2^)	23.37 ± 2.95	24.14 ± 2.91	1.10	0.97–1.24	0.15
Number of deliveries			2.09	1.30–3.37	0.002
1	104(79.4%)	197 (56.8%)			
2	26 (19.8%)	132(38%)			
3	1 (0.8%)	18(5.2%)			
Fetal weight (kg)	3.13 ± 0.56	3.25 ± 0.48	1.53	0.99–2.36	0.055

The sEMG using the Glazer protocol was employed to assess the recruitment levels of neuromuscular motor units, reflecting the functional status of the pelvic floor muscles, including muscle strength, reactivity, and endurance. There were no statistically significant differences in sEMG parameters between the DRA and non-DRA groups across all measured phases. Specifically, for the mean value of the pre-baseline phase, the difference was not statistically significant (Cohen’s *d* = 0.182, 95% CI [5.82–6.62], *p* = 0.09). For the maximum value of the flick contraction, no significant difference was observed (Cohen’s *d* = 0.03, 95% CI [38.08–41.87], *p* = 0.769). Similarly, no significant group differences were found in the mean value (Cohen’s *d* = 0.002, 95% CI [26.30–29.34], *p* = 0.808) and maximum value (Cohen’s *d* = 0.017, 95% CI [43.65–48.33], *p* = 0.757) and variability (Cohen’s *d* = 0.081, 95% CI [0.23–0.25], *p* = 0.320) of tonic contraction, as well as in the mean value (Cohen’s *d* = 0.047, 95% CI [22.64–25.29], *p* = 0.571) and variability (Cohen’s *d* = 0.077, 95% CI [0.22–0.24], *p* = 0.216) of endurance contraction, and in the mean value (Cohen’s *d* = 0.19, 95% CI [4.98–5.81], *p* = 0.105) of the post-baseline phase. For the electromyography parameters analyzed, none exhibited statistically significant differences between the Non-DRA and DRA groups after BH correction (Table 3).

**Table 3:** Comparison of the surface electromyography parameters of pelvic floor muscles between non-DRA and DRA group.

Parameters	Non-DRA group (*n* = 131)	DRA group (*n* = 347)	*t*/*Z*/*F*	Cohen’s d	*p*-value	BH-adjusted *q*-value
Pre-baseline						
mean value (uV)	5.56 ± 3.41	6.22 ± 3.82	−1.697	0.18	0.090^b^	0.180
Flick contraction						
maximum value (uV)	39.44 ± 17.94	39.98 ± 17.94	0.007	0.03	0.769^a^	0.769
Tonic contraction						
mean value (uV)	27.79 ± 14.97	27.82 ± 14.36	−2.43	0.002	0.808^b^	0.808
maximum value (uV)	45.62 ± 22.29	45.99 ± 22.18	−0.31	0.017	0.757^b^	0.757
variability	0.25 ± 0.15	0.24 ± 0.09	−0.1	0.081	0.320^b^	0.400
Endurance contraction						
mean value (uV)	23.36 ± 12.94	23.96 ± 12.59	−0.57	0.047	0.571^b^	0.571
variability	0.24 ± 0.16	0.23 ± 0.09	−1.24	0.077	0.216^b^	0.288
Post-baseline						
mean value (uV)	4.69 ± 3.40	5.40 ± 3.96	−1.62	0.19	0.105^b^	0.210

**Notes.**

a Student’s *t*-test.

b Mann–Whitney *U* test.

c Chi-square test.

d Fisher’s exact test.

*p*-value <0.05 was considered statistically significant.

*q*-value >0.05 was considered no significant differences after correction.

## Discussion

The present study revealed a 72.6% prevalence of diastasis recti abdominis at 6 weeks postpartum, which is notably higher than the 60% reported in previous research (Sperstad et al., 2016). Sperstad measured the IRD by palpation, which is not as accurate as ultrasound. Unlike prior studies that exclusively measured positions 4.5 cm above and below the umbilicus, our investigation incorporated additional measurement points at three cm above and two cm below the umbilicus. This modification was informed by previous findings indicating that the maximal separation of rectus abdominis in nulliparous women typically occurs at these locations (Beer et al., 2009). Furthermore, our study defined DRA as a separation distance exceeding two cm at any point, contrasting with the conventional criterion of greater than two fingerbreadths (approximately three cm) employed in other studies.

The risk factors of DRA are also controversial. Our analysis identified age, education level, pre-pregnancy BMI, number of deliveries, and baby birth weight as factors associated with DRA. However, these findings contradict previous research that found no association between DRA and pre-pregnancy BMI, weight gain, and baby birth weight (Fernandes et al., 2015). These inconsistencies may stem from variations in the timing of DRA assessment across studies, given the dynamic nature of DRA prevalence throughout the postpartum period. The global prevalence of DRA has been reported as 100% in the third trimester (Fernandes et al., 2015), 60% at 6 weeks postpartum, and over 30% at 1 year postpartum (Sperstad et al., 2016). Recent investigations have further elucidated these temporal patterns. Fei et al. (2021) identified cesarean delivery and multiple gestation as risk factors for DRA within one year postpartum, using an inter-recti distance (IRD) of ≥2 cm as the diagnostic threshold. Wu et al. (2021) demonstrated age-stratified risk factors, with parity and diabetes mellitus emerging as significant predictors in younger women, while obesity and diabetes were predominant in older populations A longitudinal study by Lin et al. (2024) revealed temporal variations in risk factors, with BMI emerging as significant at 10 years postpartum, parity at 3, 5, and 10 years postpartum, multiple gestation at 3 years postpartum, and diabetes mellitus at 20 and 30 years postpartum.

Interestingly, our findings revealed a statistically significant association between lower educational attainment and increased risk of DRA in the early postpartum period. Although this relationship is not widely documented, it may reflect underlying differences in health literacy, access to postpartum care, and engagement in preventive behaviors. First, lower educational levels often correlate with reduced access to high-quality prenatal and postpartum care (Gube et al., 2024). Women with limited education may receive less counseling on body mechanics, core strengthening, and pelvic floor protection strategies during pregnancy and postpartum. Second, education level is associated with nutritional quality during pregnancy, which can influence tissue integrity and recovery capacity (Yu et al., 2022). Third, those who are less well educated are most at risk of gaining weight outside of the recommendations during pregnancy contributing to DRA development (O’Brien, Alberdi & McAuliffe, 2018). Furthermore, women with lower education tend to have children at younger ages and higher parity, both of which are known risk factors for DRA (Benjamin, Van de Water & Peiris, 2014). Importantly, education may act as a proxy for broader socioeconomic status (SES), encompassing income, housing quality, social support, and access to maternal health resources. However, our study did not directly measure these variables. Additionally, we employed a binary classification of education, which may not fully capture the gradient effects of SES or subtle differences in health behaviors. These unmeasured confounding factors may have contributed to the observed association, and future studies should consider using composite SES indices or more granular educational categories. From a clinical perspective, identifying lower educational attainment as a risk marker for DRA has meaningful implications. Postpartum rehabilitation programs could integrate educational screening as part of early risk assessment and prioritize resource allocation for at-risk individuals. In summary, our finding adds to a growing body of evidence suggesting that social determinants of health—including education—play a critical role in musculoskeletal outcomes after childbirth. Further research is needed to examine the interplay between education, SES, and DRA risk, ideally through longitudinal studies incorporating diverse socioeconomic variables and behavioral mediators.

The utility of sEMG in assessing pelvic floor muscle bioelectrical activity and co-activation patterns has been well-established (Tahan et al., 2013; Chmielewska et al., 2015). Our comparative analysis of PFM sEMG parameters between DRA and non-DRA groups in the early postpartum period revealed no significant differences in PFM strength or endurance. These findings align with previous research, though it should be noted that our methodology employed ultrasound for DRA detection, which offers greater precision than the fingerbreadth measurement used in earlier studies (Wang et al., 2020). Zhang et al. (2023) found that the endurance of vaginal sphincter and external anal sphincter in DRA group was weakened compared with Non-DRA group, and the sEMG of other pelvic floor muscles showed no difference between the two groups. This discrepancy may be attributable to methodological differences, as our study assessed global PFM activity, whereas Zhang et al. evaluated individual PFM components. Surface EMG (sEMG) is a non-invasive method widely used to assess pelvic floor muscle (PFM) activity; however, it has several limitations. One major concern is the lack of signal normalization, especially in protocols such as the Glazer protocol, which can lead to substantial variability across individuals and sessions. Additionally, sEMG signals can be influenced by factors such as skin impedance, electrode placement, and cross-talk from adjacent muscles, potentially compromising measurement accuracy. Auchincloss & McLean (2009) reported that while the between-trial reliability of PFM sEMG recordings was acceptable within the same session, the test–retest reliability across different days was poor. Similarly, another study found that the between-day reliability of mean and peak amplitudes during tonic contractions, as measured using the Glazer protocol, was relatively low, further highlighting concerns regarding consistency over time (Oleksy et al., 2021).

The theory of pelvi-abdominal dynamics suggests that the abdominal and pelvic floor muscles have a synergistic effect (Sapsford et al., 2001). During pelvic floor muscle (PFM) contraction, it has been suggested that co-contraction of the abdominal muscles, particularly the transversus abdominis, may facilitate more effective pelvic floor activation (Serrano et al., 2018). Impairment of abdominal musculature may compromise this synergistic mechanism, potentially exacerbating pelvic floor dysfunction. Ptaszkowski et al. (2015) found that in menopausal women with stress urinary incontinence (SUI), when the pelvic floor muscle contracted alone, the rectus abdominis muscle contracted synergically. Recent therapeutic approaches have incorporated this understanding, with EMG-biofeedback- assisted pelvic floor muscle training combined with rectus neuromuscular electrical stimulation (NMES) was more effective than the treatment of rectus NMES alone (Liang et al., 2022). Although earlier studies raised concerns about the potential for pelvic floor muscle (PFM) and transversus abdominis contractions to transiently increase inter-rectus distance (IRD) in postpartum women, more recent evidence suggests that this effect is minimal and can be effectively counteracted by simultaneous activation of the rectus abdominis muscle. These findings highlight the importance of coordinated muscle engagement and suggest that well-designed rehabilitation protocols may mitigate any undesirable effects (Theodorsen, Strand & Bø, 2019).

In terms of the relationship between DRA and SUI, Bøet al. (2017); Braga et al. (2019); Liu et al. (2023) reported that postpartum DRA of first-time mothers was not associated with SUI, while a study by Spitznagle, Leong & Van Dillen (2007) of the prevalence of DRA in the elderly urogynecological population concluded that patients with DRA were more likely to have fecal incontinence, pelvic organ prolapse, and stress incontinence than patients without DRA. Our findings align with the former studies, showing no association between DRA and SUI during pregnancy or early postpartum. This discrepancy may reflect population differences, as Spitznagle, Leong & Van Dillen’s (2007) cohort had a median age of 52.45 years, compared to our postpartum sample. Devreese et al. (2007) conducted a study showing that the sequence of activation between superficial and deep pelvic floor muscles was consistent in continent women but altered in women with urinary incontinence. This suggests that impaired neuromuscular coordination may contribute to pelvic floor dysfunction. Future pelvic floor EMG assessments should be designed with greater precision, allowing for better differentiation of muscle layers and coordination patterns. This study was mainly to analyze the relationship between rectus abdominis muscle separation and stress incontinence, and further studies could explore the relationship and specific mechanism between rectus abdominis muscle and pelvic floor muscles in stress incontinence by detecting bioelectrical signals. At the same time, considering the synergistic effect of rectus abdominis and pelvic floor muscles, the method should be optimized to reduce the interaction between muscles in the future myoelectric assessment of pelvic floor muscles.

Several limitations should be acknowledged in this study. The retrospective design and reliance on self-reported measures for certain parameters such as physical activity and urinary incontinence may introduce recall bias. Additionally, the variability of PFM activity in the early postpartum period may have limited our ability to detect significant effects, suggesting that assessments at later time points may yield different results. Future prospective studies with standardized assessment protocols and longer follow-up periods are warranted to further elucidate the complex relationships between DRA, PFM function, and pelvic floor disorders.

Additionally, due to the retrospective design of this study, inter- and intra-rater reliability assessments could not be conducted, which may limit the consistency evaluation of the sEMG measurements. This study did not include a priori sample size calculation or power analysis, which limits the ability to determine whether the study was adequately powered to detect meaningful differences or associations. Future studies with larger sample sizes and confirmatory analyses are needed to validate these findings and reduce the likelihood of false positives. Otherwise, the participants were recruited from a single tertiary hospital within a 3-month period, which may not fully represent the broader postpartum population. Women attending a tertiary center may differ in demographic or clinical characteristics from those in primary or community settings, potentially limiting the generalizability of our findings. Therefore, the results should be interpreted with caution, and confirmation in larger, multi-center studies with more diverse populations and longer recruitment windows is warranted.

## Conclusions

This retrospective study identified several factors independently associated with the presence of diastasis recti abdominis in the early postpartum period. Multivariate logistic regression analysis showed that older maternal age, lower educational attainment, and higher parity were significantly associated with an increased risk of DRA. In contrast, factors such as fetal weight, pre-pregnancy BMI, and postpartum BMI were not significant in the final model. Additionally, no association was found between pelvic floor muscle function and the presence of DRA. These findings suggest that the clinical management of postpartum DRA should prioritize targeted abdominal and core rehabilitation strategies rather than focusing exclusively on PFM strengthening exercises. This approach aligns with the current understanding of abdominal-pelvic dynamics and may provide more effective outcomes in the treatment and prevention of postpartum DRA.

##  Supplemental Information

10.7717/peerj.20299/supp-1 Supplemental Information 1 Raw data

10.7717/peerj.20299/supp-2 Supplemental Information 2 STROBE checklist

## Additional Information and Declarations

### Data Availability

The following information was supplied regarding data availability: The raw measurements are available in the Supplementary File.
