# Supplementary material for: Risk factors of diastasis recti abdominis, and relationship with surface electromyography characteristics of pelvic floor muscles in early postpartum: a retrospective study
Source: PeerJ. 2025 Nov 7;13:e20299. doi: 10.7717/peerj.20299 (PMC12599372; doi:10.7717/peerj.20299)
Supplement: Supplemental Information 2 [file peerj-13-20299-s002.pdf]

STROBE Statement—Checklist of items that should be included in reports of ***case-control studies***

	Item No	Recommendation
**Title and abstract**	1☑	(*a*) Indicate the study's design with a commonly used term in the title or the abstract **(line 3)**
		(*b*) Provide in the abstract an informative and balanced summary of what was done and what was found **(methods and results)**
**Introduction**		
Background/rationale	2☑	Explain the scientific background and rationale for the investigation being reported **(line 19-52)**
Objectives	3☑	State specific objectives, including any prespecified hypotheses **(line 54-61)**
**Methods**		
Study design	4☑	Present key elements of study design early in the paper **(abstract-methods)**
Setting	5☑	Describe the setting, locations, and relevant dates, including periods of recruitment, exposure, follow-up, and data collection **(participants)**
Participants	6☑	(*a*) Give the eligibility criteria, and the sources and methods of case ascertainment and control selection. Give the rationale for the choice of cases and controls **(participants)**
		(*b*) For matched studies, give matching criteria and the number of controls per case **(inapplicability)**
Variables	7☑	Clearly define all outcomes, exposures, predictors, potential confounders, and effect modifiers. Give diagnostic criteria, if applicable **(line 73-83)**
Data sources/ measurement	8*☑	For each variable of interest, give sources of data and details of methods of assessment (measurement). Describe comparability of assessment methods if there is more than one group **(Methods)**
Bias	9☑	Describe any efforts to address potential sources of bias （**Statistical analysis**）
Study size	10	Explain how the study size was arrived at<br>**Note：This is a retrospective analysis without sample size estimates**
Quantitative variables	11☑	Explain how quantitative variables were handled in the analyses. If applicable, describe which groupings were chosen and why **（Statistical analysis）**
Statistical methods	12☑	(*a*) Describe all statistical methods, including those used to control for confounding **(Statistical analysis)**
		(*b*) Describe any methods used to examine subgroups and interactions**(inapplicability)**
		(*c*) Explain how missing data were addressed**(inapplicability)**
		(*d*) If applicable, explain how matching of cases and controls was addressed**(inapplicability)**
		(*e*) Describe any sensitivity analyses**(inapplicability)**
**Results**		
Participants	13*	(a) Report numbers of individuals at each stage of study—eg numbers potentially eligible, examined for eligibility, confirmed eligible, included in the study, completing follow-up, and analysed
		(b) Give reasons for non-participation at each stage
		(c) Consider use of a flow diagram<br>**Note: This is a retrospective study analysis, with participants automatically selected from the database to meet the criteria.**

Descriptive data	14*☑	(a) Give characteristics of study participants (eg demographic, clinical, social) and information on exposures and potential confounders **(Table 1)**
		(b) Indicate number of participants with missing data for each variable of interest (none)
Outcome data	15*☑	Report numbers in each exposure category, or summary measures of exposure **(line 112-116)**
Main results	16☑	(*a*) Give unadjusted estimates and, if applicable, confounder-adjusted estimates and their precision (eg, 95% confidence interval). Make clear which confounders were adjusted for and why they were included **(inapplicability)**
		(*b*) Report category boundaries when continuous variables were categorized **(Table 1)**
		(*c*) If relevant, consider translating estimates of relative risk into absolute risk for a meaningful time period **(inapplicability)**

Other analyses	17	Report other analyses done—eg analyses of subgroups and interactions, and sensitivity analyses<br>**(inapplicability)**
**Discussion**		
Key results	18☑	Summarise key results with reference to study objectives **(line 134,145,198)**
Limitations	19☑	Discuss limitations of the study, taking into account sources of potential bias or imprecision. Discuss both direction and magnitude of any potential bias
Interpretation	20☑	Give a cautious overall interpretation of results considering objectives, limitations, multiplicity of analyses, results from similar studies, and other relevant evidence **(discussion)**
Generalisability	21☑	Discuss the generalisability (external validity) of the study results**(discussion)**
**Other information**		
Funding	22☑	Give the source of funding and the role of the funders for the present study and, if applicable, for the original study on which the present article is based **(funding statement)**

*Give information separately for cases and controls.

Note: An Explanation and Elaboration article discusses each checklist item and gives methodological background and published examples of transparent reporting. The STROBE checklist is best used in conjunction with this article (freely available on the Web sites of PLoS Medicine at http://www.plosmedicine.org/, Annals of Internal Medicine at http://www.annals.org/, and Epidemiology at http://www.epidem.com/). Information on the STROBE Initiative is available at http://www.strobe-statement.org.
